# Implicit versus explicit processing of visual, olfactory, and multimodal landmark information in human wayfinding

**DOI:** 10.3389/fpsyg.2023.1285034

**Published:** 2023-11-15

**Authors:** Mira Schwarz, Kai Hamburger

**Affiliations:** Department of Experimental Psychology and Cognitive Science, Faculty of Psychology and Sport Science, Justus Liebig University Giessen, Giessen, Germany

**Keywords:** wayfinding, recognition, implicit processing, olfaction, spatial cognition

## Abstract

Despite the predominant focus on visual perception in most studies, the role of humans’ sense of smell in navigation has often been neglected. Recent research, however, could show that humans are indeed able to use their sense of smell for orientation, particularly when processed implicitly. In this study, we investigate whether implicit perception of olfactory landmarks enhanced wayfinding performance compared to explicit perception. Fifty-two people completed a wayfinding and a recognition task in a virtual maze at two times of testing 1 month apart. Participants either received olfactory, visual, or both cues at the intersections. Wayfinding performance was better for olfactory landmarks, which were not correctly remembered in the recognition task. In contrast, wayfinding performance was better when visual landmarks were correctly remembered. In the multimodal condition, wayfinding performance was better with landmarks being remembered at t1 and remained the same at t2. Our results suggest distinct implicit processing mechanisms within the olfactory system and therefore hold important implications for the nature of spatial odor processing extending beyond explicit odor localization tasks. The study highlights the importance for future studies to develop and employ further experimental methods that capture implicit processing across all of our senses. This is crucial for a comprehensive understanding of consciousness, as olfaction strongly influences our behavior, but remains largely latent unless deliberately honed through practice.

## 1. Introduction

The use of odors to influence human behavior is widespread in practices like aromatherapies (Tisserand and Balacs, 1996) and marketing (Emsenhuber, 2009). The impact of olfactory information on our behavior is commonly accepted in society (Degel and Köster, 1999). Nevertheless, to date there has been limited research studying this influence, as research has long credited the human olfactory system with only its classical functions for self-preservation [finding food (Yeomans, 2006) or perceiving warning signals (Scherer and Quast, 2001)]. For a long time, humans were even considered anosmatic, as suggested by Broca (1879). Looking back, this wasn’t due to their lack of olfactory abilities, but rather their inability to consciously select a response to an olfactory stimulus (McGann, 2017). However, over time, distinct features of human olfaction were discovered that distinguish it from all other senses. With its uniqueness, the sense of smell thus represents a meaningful approach for future research into fundamental human brain processes.

One of the outstanding features of the human olfactory system is its close connection to our emotions (Aggleton and Mishkin, 1986). This connection, which was initially based only on introspection and observation, received tremendous support from brain imaging studies (Aggleton and Mishkin, 1986; Herz, 1998; Herz et al., 2004); and could potentially explain why the sense of smell plays a fundamental role in shaping our behavior, given that emotions have a pervasive influence on virtually every aspect of cognition (Tyng et al., 2017).

Moreover, the olfactory system exhibits a unique connection with memory (Stäubli et al., 1984; Schwerdtfeger et al., 1990; Cahill et al., 1995; White et al., 2015). In particular, long-term memory displays exceptional resistance to decay, while Herz and Engen (1996) found short-term memory to be relatively weak or even absent. However, since they tested short-term memory only explicitly, its absence could also imply that the odors were not processed explicitly, but rather implicitly. While explicit memory involves conscious recall, as required for example in a vocabulary test; implicit memory is used unconsciously (Buchner and Wippich, 1998). Classic examples for that include riding a bike or reading a book. Both memory systems influence our behavior in everyday life (more or less consciously). While there are many studies on explicit memory, it is difficult to study implicit memory. Especially implicit olfactory memory remains largely unexplored and has only partly been demonstrated to date (e.g., Schab and Crowder, 2014). However, Degel and Köster’s (1998, 1999) pioneering studies on implicit olfactory memory revealed evidence of implicitly learned odor memories. Participants rated odor congruence with visual contexts, showcasing an early systematic exploration of implicit olfactory memory (Degel and Köster, 1998, 1999). Exposure to an odor unknowingly resulted in later association of the odor with its exposure site. Interestingly, this effect manifested when participants could not label the unconsciously perceived odor. Naming the odor could impede implicit memory, indicating that odor naming might negatively impact wayfinding. This finding was also confirmed in a repetition priming experiment with odors by Olsson (1999). He demonstrated that incorrectly identified odors were processed faster than odors that were correctly identified. This finding again provides evidence for a possible interference effect of explicit processing of odors (i.e., knowing the name of an odor) with the establishment, retention, or retrieval of (implicit?) odor memory (see also Degel et al., 2001). Moreover, Moessnang et al. (2011) used a directional smell cueing paradigm, indicating implicit directional smelling ability. Olfactory stimuli congruent with cued targets led to slower responses, highlighting cross-modal attentional interference. The explicit condition performance was at chance-level, showing humans’ incapability to consciously determine odor location. Wudarczyk et al. (2016) adopted Moessnang et al.’s (2011) paradigm to investigate implicit and explicit processing differences of olfactory and trigeminal stimuli, supporting an implicit-explicit dissociation of olfactory localization (Wudarczyk et al., 2016).

Besides its distinctive connection to emotions and memory, the sense of smell possesses another unique trait: it phylogenetically stands as the oldest sense, being the initial form of interorganism communication (Hoover, 2010). Looking at the evolution of the vertebrate brain, olfactory bulb size shows unparalleled variability, distinct from other brain regions scaling with brain size (Jacobs, 2012). Although this variability appears to be a consequence of olfactory functions, it remains unexplained within the classical olfactory functions (see above). This finding prompted Jacobs (2012) to propose the *olfactory spatial hypothesis*, suggesting that the sense of smell originally evolved to support spatial orientation (Dahmani et al., 2018; Jacobs, 2019), thereby significantly influencing perception and navigation (Huber et al., 2022). If navigation underpins olfaction’s primary role (i.e., predicting odorant distributions in time and space) – instead of self-preservation in terms of finding food or perceiving warning signals - olfactory bulb size variation effectively reflects the navigational demands of different vertebrate species (Jacobs, 2012). Following this hypothesis, new discoveries caused many of our preconceived notions about the contribution of olfaction to spatial representations to be challenged (Jacobs, 2022).

In contrast to prior beliefs, where navigation was primarily viewed as a visual process and research largely focused on unimodal visual wayfinding, the olfactory spatial hypothesis promoted experiments demonstrating that humans are also able to navigate through (virtual) environments based on their sense of smell alone (Jacobs et al., 2015; Hamburger and Knauff, 2019; Schwarz and Hamburger, 2022). Remarkably, wayfinding performance did not differ between different modalities (i.e., auditory, visual, verbal, olfactory; Hamburger and Röser, 2014; Arena and Hamburger, 2023). In these experiments, so-called *landmarks* were used as orientation reference. The existing literature defines landmarks as distinct objects or location in an environment that serves to define the location (Hirtle, 2008). Even though, it seems intuitively plausible that mainly visual landmarks are incorporated for the construction of cognitive maps, we propose a multimodal representation of cognitive maps in which our senses work together rather than acting as separate entities (Karimpur and Hamburger, 2016; Hamburger and Knauff, 2019; Arena and Hamburger, 2022; Schwarz and Hamburger, 2022). Despite a widespread acceptance of multimodal sensory processing (e.g., Spence, 2020), research mainly remains unimodal for human navigation. We believe that to comprehensively understand human cognition, we urge to shift from a unimodal perspective to a more realistic multimodal comprehension, especially in the context of spatial cognition.

With regard to olfactory spatial cognition, humans tend to use olfactory landmarks implicitly rather than explicitly in navigation (Moessnang et al., 2011; Wudarczyk et al., 2016). This poses two major issues: studies often omit smell due to exclusive reliance on explicit processing methods, such as recognition tasks only (Abu-Obeid, 1998; Choi et al., 2016); second, data from experiments that capture the implicit olfactory processing cannot yet be explained by existing theories because processing odor stimuli is still poorly understood. However, previous wayfinding studies involving olfactory landmarks already yielded contradictory results regarding performance in recognition and wayfinding tasks. While recognition outperformed wayfinding across all sensory modalities by around 10% (Hamburger and Röser, 2014; Karimpur and Hamburger, 2016; Arena and Hamburger, 2022), intriguingly, incorrectly recognized olfactory landmarks still facilitated accurate wayfinding decisions (Arena and Hamburger, 2022). This phenomenon was exclusive to olfactory landmarks, implying that recognition is not a prerequisite for effective wayfinding using odors and stands in line with previous findings regarding implicit odor memory (see above). Furthermore, investigations into “switching costs” - the cognitive toll of switching modalities during tasks – indicated no decline in wayfinding performance when alternating between auditory and visual landmarks (Hamburger and Röser, 2011). This aligns with the idea that images and sounds engage the same cognitive system. Conversely, transitioning between olfactory and visual landmarks incurred switching costs and wayfinding performance reduction (Schwarz and Hamburger, 2022), pointing toward distinct cognitive processing for odors and images. An explanation for that could be an implicit use of olfactory landmark information in comparison to an explicit use of visual landmarks. Here, Hamburger (2020) applies the cognitive concept of two processing systems (system 1 for fast, automatic processing and system 2 for conscious, deliberate processing; Kahneman, 2011) to landmark-based wayfinding. In an unfamiliar environment, conscious landmark-use engages System 2, whereas familiar environments likely trigger unconscious, System 1-based processing. We assume, olfactory landmarks likely engage the evolutionary older System 1, given our inability to consciously perceive or name most odors.

In summary, the olfactory system is essential for wayfinding in many mammals (e.g., Steck, 2012) and almost certainly evolved originally in humans to support spatial navigation (e.g., Dahmani et al., 2018; Jacobs, 2019). We are therefore indeed capable of using olfaction for wayfinding (e.g., Hamburger and Knauff, 2019), but it is believed that this is only possible when assessed implicitly (e.g., Moessnang et al., 2011). Due to these numerous peculiarities of the human olfactory system, the sense of smell is an excellent model for investigating implicit, emotional, sensory processing and especially human navigation and orientation processing. Despite the visual sense remaining the most important of all senses in human navigation, we want to focus on a more comprehensive understanding of navigation with all senses. Therefore, this study tests whether implicit processing of olfactory landmark information leads to better wayfinding performance than explicit processing of olfactory landmarks. We aim to show that olfactory landmarks are not required to be consciously perceived to ensure successful wayfinding.

## 2. Materials and methods

### 2.1. Participants

Fifty-two people volunteers partook in the experiment at both times of testing, divided pseudo-randomly across three conditions (39 female, 13 male; age: 19–61, *M* = 27.27, *SD* = 11.11; olfactory unimodal: *n* = 16, visual unimodal: *n* = 18, visual × olfactory multimodal: *n* = 18). As already demonstrated in Hamburger and Röser (2014) no gender differences were found in the current study. All participants had normal olfactory and visual functions, and their written informed consent was obtained, approved by a local ethics committee (Department of Psychology, JLU; 2014-0017) adhering to the Declaration of Helsinki guidelines. Participants received course credits or entered a raffle for ten 20€ Amazon vouchers as compensation.

### 2.2. Material

**Figure 1:**
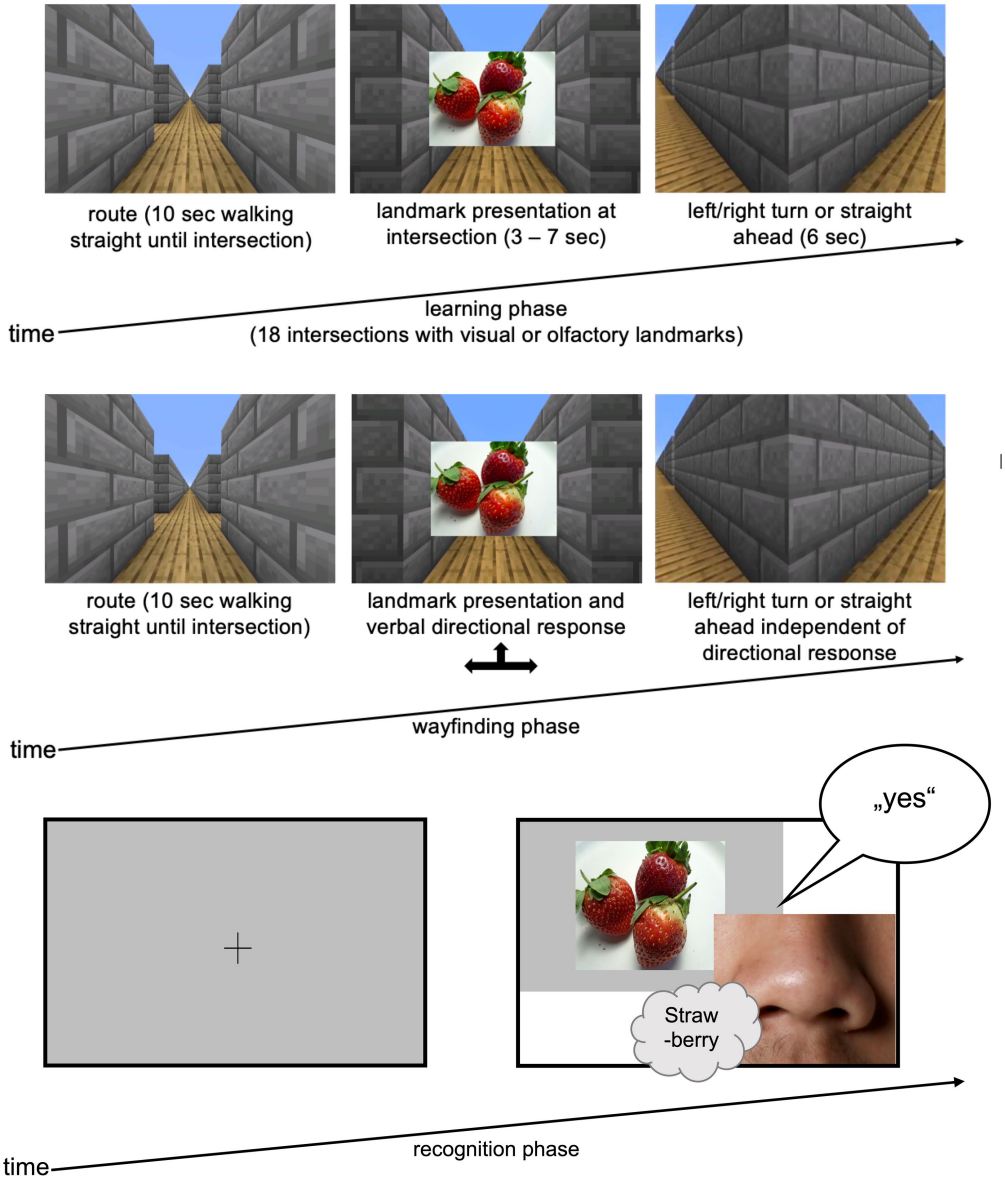
Experimental procedure. (left) Exemplary route for the *learning phase* in a virtual environment built with Minecraft® with either olfactory or visual cues at each intersection (cloud symbol); (top right) in the *wayfinding phase* participants again saw the video sequence which was stopped at every intersection and they had to decide – based on the specific landmark (olfactory or visual) at the intersection – whether to turn right, left or move straight; (bottom right) in the *recognition phase* the 18 landmarks plus 18 distractors (olfactory or visual) were presented in random order, participants had to decide whether they already smelled/saw the cues in the learning and wayfinding phase (“yes”/“no”).

Data collection comprised three experimental blocks: *learning-, wayfinding-and recognition phases* (Figure 1). Throughout, participants wore an HTC Vive head-mounted display (HMD) to minimize distractions and ensure equal immersion for all participants. A video showed a path through a self-built 3D virtual maze created with Minecraft® (Mojang Synergies AB, n.d.). The route, shared among all conditions, included 12 directional changes (six right, six left) and passed straight ahead six times at a total of 18 intersections. To counter potential position biases, half the participants viewed the vertically mirrored video, reversing turns systematically. At each intersection, participants encountered an odor, picture, or both (= *landmarks*).

Olfactory landmarks were selected based on an olfactory salience assessment (valence, arousal, dominance) by Hamburger and Herold (2021). We used 36 odors with varying valence, arousal, and dominance. From these, 18 odor pairs of similar valence scores (according to Hamburger and Herold, 2021) were created, with half serving as landmarks. The remaining 18 matched odors were distractors for the recognition task, appearing randomly alongside the 18 landmarks. For visual and multimodal conditions, 36 photos matching the chosen odors (e.g., strawberry scent and strawberry image) were gathered from private sources and the license-free stock images provider pexels.com. Therefore, half the odors and matching pictures (*n* = 18) functioned as distractors, while the rest became visual and olfactory landmarks.

A comprehensive list of landmarks and distractors is available in the Supplementary Material.

### 2.3. Procedure

In the first experimental block, the *learning phase*, participants watched the video of the route through the virtual maze including 18 landmarks (visual, olfactory or both) at intersection. Their task was to remember the route after a single viewing. Landmarks were presented as pictures at intersection for 3 s (= visual condition; Figure 1) or as hand-administered odors for 7 s (= olfactory condition) since the processing time of odors is longer than for pictures (Cain, 1976; Posner and Cohen, 1984). In the multimodal condition, participants watched the video with visual landmarks while simultaneously receiving matching olfactory landmarks by hand for 7 s. See Figure 1 for further time specifications.

For the *wayfinding task*, the second experimental phase, the same video was presented. At intersections, the video paused until the participants verbally and or manually by hand signal indicated the route direction (left, right, straight), allowing for possible left–right confusion. Regardless of their response, the video continued into the correct direction. This allowed participants to check whether they answered correctly (i.e., if the route in the video continued in the same direction as they indicated) or if they made a mistake (i.e., if the route continued in a different direction than indicated).

The final experimental block, the *recognition task*, presented 18 landmarks and 18 distractors in randomized order, using the same modality as in the learning phase. Participants swiftly identified if the presented stimulus was a landmark or distractor, responding verbally with “yes” or “no.” To counter position biases, six randomized stimulus representations were created for the recognition task.

One month later, the second testing (t2) excluded the learning phase, focusing on experimental blocks 2 and 3. Route, stimulus sequence, and modality were consistent with the first testing (t1). After these tasks, participants completed a questionnaire covering demographics, strategy usage, and prior experiences with olfactory and navigation experiments.

Repeated and prolonged presentation of similar odors results in olfactory adaptation, which can cause perceptual decrease (Ferdenzi et al., 2014). With their outstanding, intense smell, coffee beans can have the ability to avoid this olfactory fatigue. Therefore, throughout the experiment, participants in the olfactory and multimodal conditions could pause to reset their olfactory sensitivity by smelling coffee beans, ensuring sustained olfactory discrimination ability (Secundo and Sobel, 2006). A minority of participants used coffee beans to neutralize their sense of smell only during the recognition phase. In the wayfinding phase, the intervals between landmark presentation at each intersection were long enough (16 s, see Figure 1) to avoid olfactory adaption. Pausing had no effect on recognition performance.

## 3. Results

### 3.1. Data reconstruction

For interference analyses, we categorized participants’ wayfinding performance into *explicit* and *implicit (?) processing* groups.

In the experiment, participants recalled directions at intersections for the wayfinding task, while the recognition task explicitly focused on correct landmark recall without linking it to path decisions. Recognition tests are direct memory tests, measuring explicit memory whereas the wayfinding task cannot yet be clearly classified as an implicit or explicit memory test. If a participant correctly identified a landmark in the recognition phase, we inferred that this landmark had been explicitly processed in the previous wayfinding phase and that the participant could therefore identify it in the subsequent recognition phase. This created an explicit processing subgroup, containing wayfinding responses for intersections where participants correctly recognized corresponding landmarks.

However, during the experiment and data review, we noted instances where landmarks were often not correctly recognized in the recognition phase yet still led to accurate wayfinding decision in the preceding wayfinding phase. This was also reflected in the participants’ comments during the experiment, as they verbally told the experimenter that they no longer had any memory of the route or landmarks, especially at the second time of testing. Nevertheless, the wayfinding performances were above chance level. This finding is particularly noteworthy considering the chance probabilities of correct responses. The chance of randomly giving a correct answer is 50% in recognition (two options: landmark vs. distractor) and one-third in wayfinding (three options: right, left, straight). It is therefore striking that correct recognition responses by chance were likelier than wayfinding responses.

If a landmark cannot be explicitly recalled in recognition yet, it still leads to accurate wayfinding; reasons could be (1) encoding failure, (2) retrieval failure, (3) pure luck in giving the correct wayfinding response, (4) sequential learning of the route in the wayfinding task, or (5) exclusive implicit processing of this landmark. In any previous experiments using the same wayfinding and recognition tasks (e.g., Hamburger and Knauff, 2019), sequential learning, instead of landmark-based learning, was always controlled for. This was tested by additional control conditions such as a task where participants were “beamed” to different intersections where landmarks were again presented without walking the route. Participants then had to verbally indicate the route direction. The wayfinding performance during the beaming phase was equal to the initial wayfinding phase, indicating no sequential learning. If sequential learning had occurred, participants would not have been able to answer correctly in the beaming phase. This approach has already been used frequently in wayfinding studies using similar wayfinding tasks, consistently yielding the same results (Balaban et al., 2014; Hamburger and Röser, 2014; Karimpur and Hamburger, 2016). Moreover, if wayfinding performance was primarily due to sequential learning rather than landmark-based wayfinding, participants would only remember the sequence of directions (i.e., “left,” “right,” “straight,” “left,” …), with little recollection of the presented landmarks. Consequently, if sequential learning were the dominant factor, recognition performance would be expected to be worse than wayfinding performance, since only the directions could be recalled. However, this is not the case, as recognition performance exceeds wayfinding performance across all three landmark modalities. This was also the case in the previous experiments where we additionally controlled for sequential learning. Nonetheless, it is important to note that sequential learning cannot be entirely ruled out for all participants at all intersections. Hamburger (2020) argues that landmark-based wayfinding in everyday life likely involves a combination of sequential learning and landmark knowledge. Nevertheless, in the present sample, based on the aforementioned reasons, it can be concluded that sequential learning played a minor role, while landmark knowledge was the dominant factor.

Hence, the second subgroup includes wayfinding responses where participants did not correctly recognize landmarks in the recognition phase. For example, if a participant correctly responded directionally for the “strawberry” landmark in wayfinding but incorrectly responded in the subsequent recognition task, implicit processing might have occurred. While the landmark was not explicitly remembered, it still led to accurate wayfinding. Moreover, the second subgroup also contained incorrect wayfinding responses, e.g., when a participant made errors for the landmark “fish” in both the wayfinding task and the recognition task. In the following paragraphs, the second subgroup is referred to as the “implicit processing” group. However, this expression must be used and interpreted with caution, since we cannot clearly exclude that encoding failure, retrieval failure, pure luck or sequential learning were the reason for recognition difficulties of landmarks. For further information and details, please see Section 4.1.

To clarify our approach, we did not calculate performance comparisons between participants but focused on comparisons between individual landmarks. Therefore, our following analyses use a data set which does not consist of just one data point per participant; instead, it includes 36 data points, corresponding to the 18 landmarks at both times of testing (18 × 2). For example, within a single participant, 12 landmarks might be categorized as “implicitly processed,” while the remaining 24 are considered “explicitly processed.”

This methodology allowed us to avoid splitting the 52 experimental subjects into numerous subgroups.

### 3.2. Interference statistics

Data were analyzed using IBM SPSS, version 28.0 (IBM Corp, 2021). For all results, significances, as well as effect sizes are reported. The test assumption of normal distribution tested with Kolmogorov–Smirnov–tests was given for all conditions at all times. Further, Levene tests showed equal variances for most of the conditions. In case of unequal variances Welch’s *t*-tests are reported. All reported *t*-tests are for independent samples and are Bonferroni corrected. Wayfinding performance was assessed as percentage of correct route decisions. For this purpose, the number of correct wayfinding decisions was divided by 18 (number of interactions) and multiplied by 100.

We first calculated a three-way ANOVA with the two between-subject factors “modality” (olfactory, visual, or multimodal) and “processing” (implicit or explicit) and the within-subject factor “time” (first time of testing and second time of testing 1 month later). All interaction effects between the three factors were significant [time × modality: *F*(2, 66) = 15.163, *p* < 0.001, *η* = 0.315; time x processing: *F*(1, 66) = 5.673, *p* = 0.020, *η* = 0.079; modality × processing: *F*(2, 66) = 10.255, *p* < 0.001, *η* = 0.237; time × modality × processing: *F*(2, 66) = 8.448, *p* < 0.001, *η* = 0.204].

**Figure 2:**
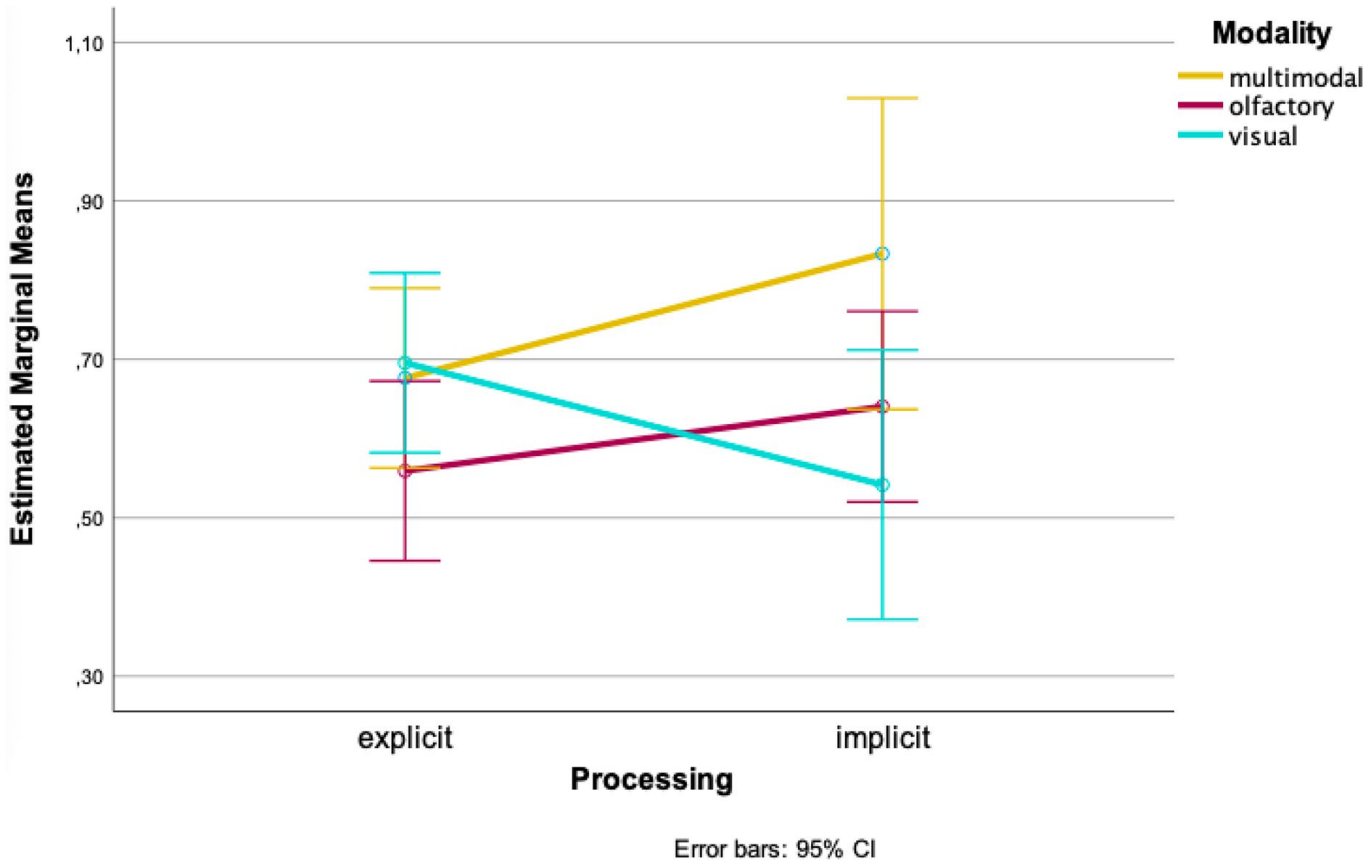
Mean wayfinding performance for the first time of testing.

When looking at the mean values of the groups separately, higher wayfinding performances in the olfactory and multimodal conditions were found for implicit processing of landmarks compared to explicit processing at the first time of testing [olfactory: *M_explicit_* = 0.559, *SD_explicit_* = 0.177, *M_implicit_* = 0.640, *SD_implicit_* = 0.274; *t*(32) = −1.039, *p* = 0.153, *d* = 0.228; 95%-CI (−0.241, 0.078); multimodal: *M_explicit_* = 0.676, *SD_explicit_* = 0.104, *M_implicit_* = 0.833, *SD_implicit_* = 0.408, *t*(5.217) = −0.932, *p* = 0.196, *d* = 0.215; 95%-CI (−0.585, 0.271); Figure 2]. The visual condition, on the other hand, showed higher values in explicit processing [*M_explicit_* = 0.696, *SD_explicit_* = 0.084, *M_implicit_* = 0.542, *SD_implicit_* = 0.502, *t*(7.173) = 0.862, *p* = 0.208, *d* = 0.280; 95%-CI (−0.266, 0.574); Figure 2].

**Figure 3:**
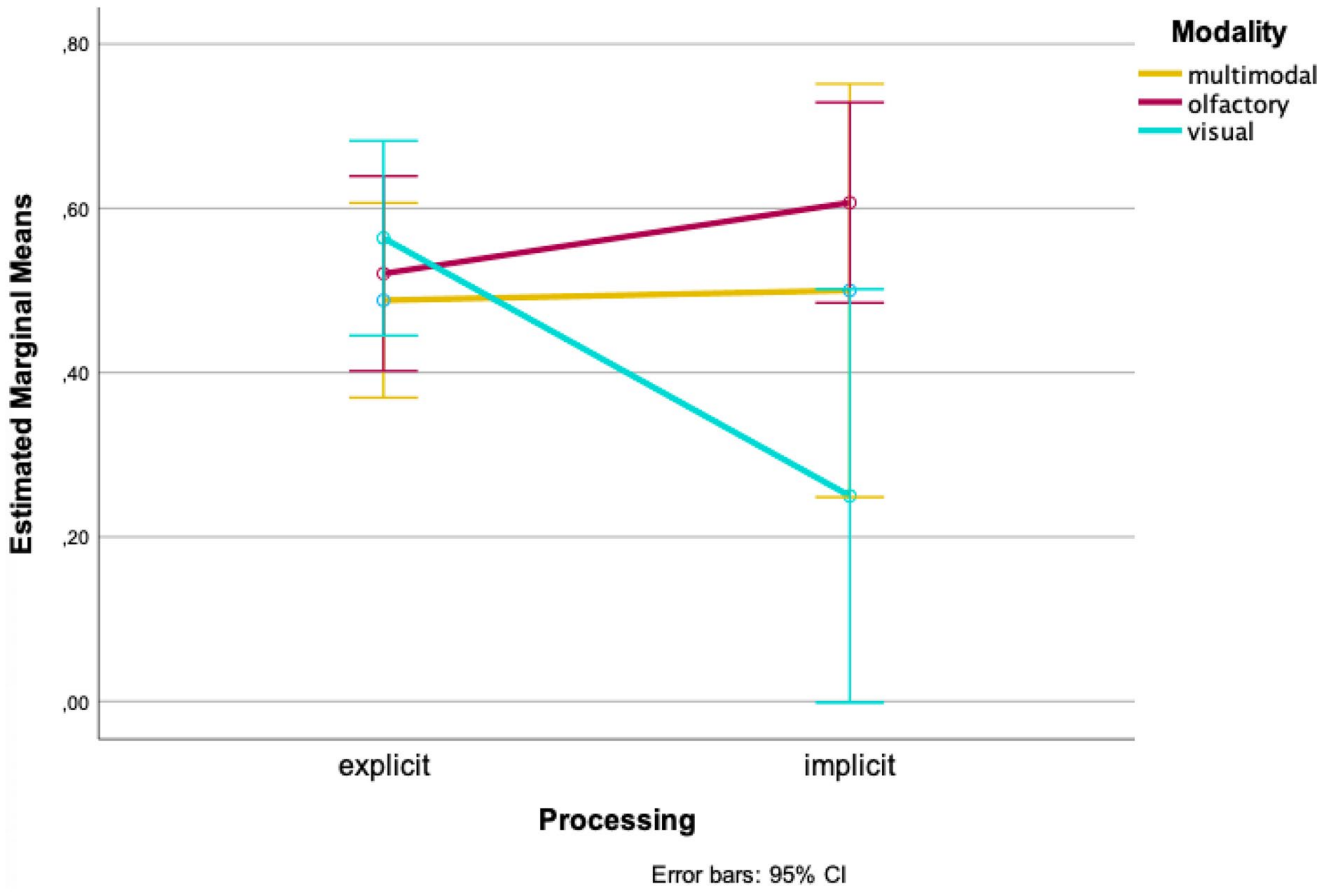
Mean wayfinding performance for the second time of testing.

At the second time of testing, the wayfinding performance in the multimodal condition is almost equally good for explicit and implicit processing [*M_explicit_* = 0.488, *SD_explicit_* = 0.157, *M_implicit_* = 0.500, *SD_implicit_* = 0.577, *t*(3.099) = −0.041, *p* = 0.485, *d* = 0.266; 95%-CI (−0.922, 0.898); Figure 3]. Once again, at the second time of testing wayfinding performance of the olfactory condition is higher for implicit processing [*M_explicit_* = 0.521, *SD_explicit_* = 0.191, *M_implicit_* = 0.607, *SD_implicit_* = 0.301; *t*(33) = −1.020, *p* = 0.158, *d* = 0.250; 95%-CI (−0.259, 0.086); Figure 3]. In the visual condition wayfinding performance was higher when processing explicitly at the second time of testing [*M_explicit_* = 0.564, *SD_explicit_* = 0.168, *M_implicit_* = 0.533, *SD_implicit_* = 0.388, *t*(3.130) = 1.242, *p* = 0.150, *d* = 0.241; 95%-CI (−0.472, 1.100); Figure 3].

**Figure 4:**
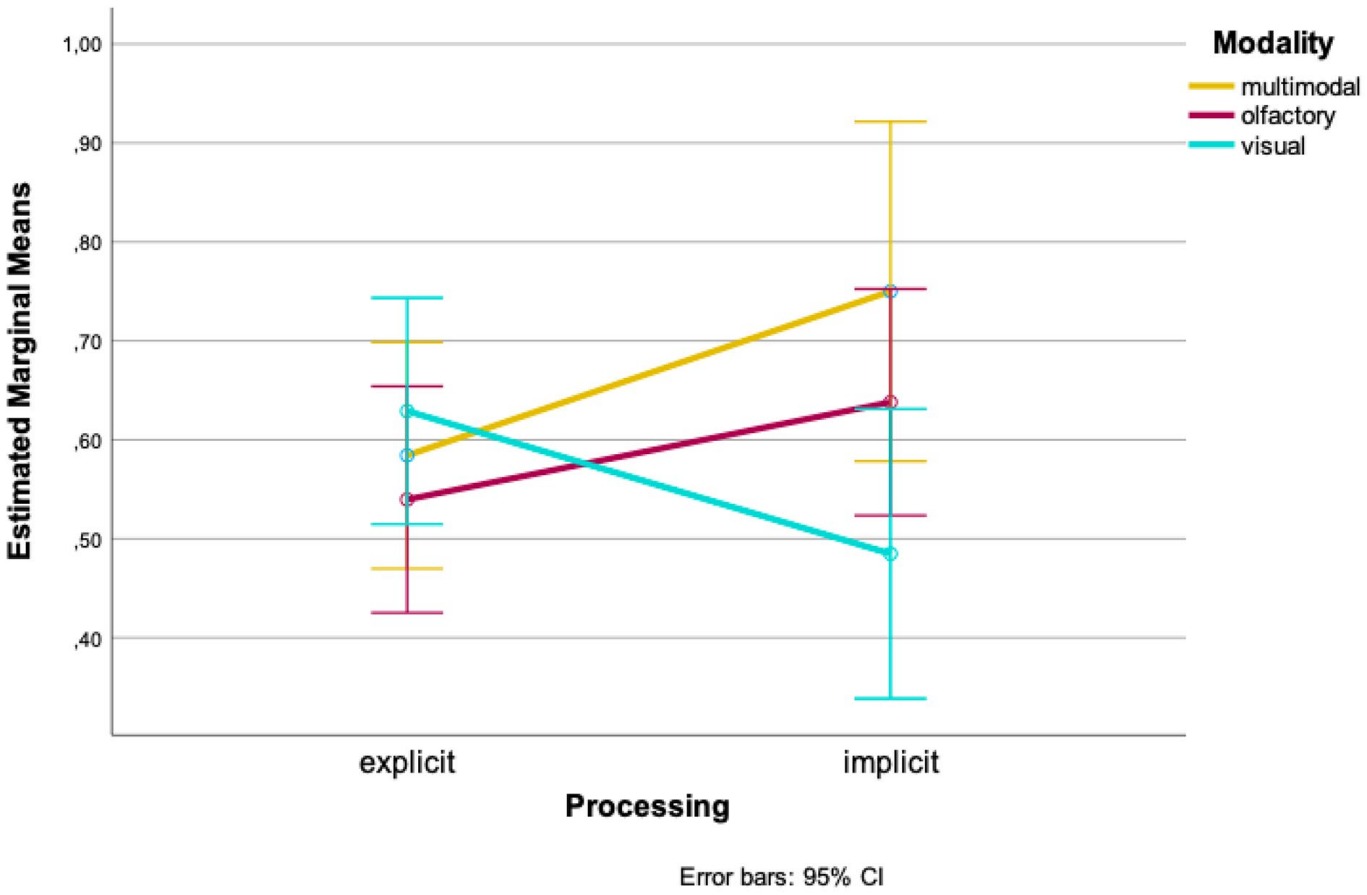
Mean wayfinding performance for both times of testing.

In further analyses, we looked at the data from both times of testing combined. A two-factor ANOVA with the between-subject factors “modality” and “processing” revealed a marginally non-significant interaction of both conditions [*F*(2, 85) = 2.936, *p* = 0.059, *η* = 0.065]. Looking at the mean values of the groups separately, higher wayfinding performances in the olfactory and multimodal conditions were found for implicit processing of landmarks compared to explicit processing [olfactory: *M_explicit_* = 0.540, *SD_explicit_* = 0.161, *M_implicit_* = 0.638, *SD_implicit_* = 0.202; *t*(34) = −1.617, *p* = 0.058, *d* = 0.182; 96%-CI (−0.222, 0.025); multimodal: *M_explicit_* = 0.584, *SD_explicit_* = 0.116, *M_implicit_* = 0.750, *SD_implicit_* = 0.378, *t*(7,595) = −1.215, *p* = 0.130, *d* = 0.226; 95%-CI (−0.483, 0.152); Figure 4]. Looking at the visual condition, an opposite effect was found: wayfinding performance was higher when landmarks were processed explicitly whereas it was lower when they were processed implicitly [*M_explicit_* = 0.629, *SD_explicit_* = 0.102, *M_implicit_* = 0.485, *SD_implicit_* = 0.503, *t*(10,511) = 0.940, *p* = 0.184, *d* = 0.316; 95%-CI (−0.195, 0.484); Figure 4].

## 4. Discussion

The question of whether and how humans are also able to orient themselves using their sense of smell has long been neglected in research on spatial thinking. The present study aimed to contribute to this field of research by investigating the implicit spatial processing of two sensory systems and its interaction. By using a recognition and wayfinding task to assess explicit and implicit processing of visual, olfactory and multimodal (visual × olfactory) cues, our results point to a facilitation of wayfinding performance by implicit processing of olfactory cues.

Our analyses show interaction effects of the variables “time of testing,” “processing” and “modality.” When looking at the means, wayfinding performance was better for olfactory landmarks, which were not correctly remembered in the recognition task compared to when correctly remembered. In contrast, wayfinding performance was better when visual landmarks were correctly remembered compared to when not correctly remembered. In the multimodal condition, at the first time of testing, wayfinding performance was better with landmarks being remembered; at the second time of testing wayfinding performance remained almost the same. However, all pairwise comparisons did not become significant.

With that, the present study is the first to differentiate between implicit and explicit processing of the participants’ wayfinding performance. And, it reveals a finding consistent with our hypothesis of implicit processing: People seem to be very good in navigating when using olfactory stimuli as landmarks without explicitly memorizing them in a subsequent recognition task, whereas visual landmarks are mainly used explicitly. Outside the laboratory setting, people are likely to use all modalities available to them implicitly or explicitly (depending on the modality). Here, it seems as if the wayfinding performance in the multimodal condition could be derived additively from olfactory and visual performance since visual performance is worse with implicit processing, but wayfinding performance is better with implicit olfactory processing. Especially at the second time of testing, these two opposite effects of the unimodal conditions seem to balance out in the multimodal condition, as wayfinding performance remains the same for implicit and explicit processing.

We assume that the ability to navigate through the virtual maze relies on short-term memory at the first time of testing whereas at the second time of testing the wayfinding task must be solved using long-term memory. According to previous studies, there seems to be hardly any or even no (explicit) short-term memory for odors (Herz and Engen, 1996). Therefore, explicit performance in the multimodal condition must rely mainly on visual information, which is shown by the almost equal performance of the visual and multimodal explicit condition at first time of testing (while the olfactory explicit performance is worse). Apparently, when implicit processing is involved in the multimodal condition, both implicit olfactory and visual information is available to the participants, which could additively lead to a better performance in the multimodal condition than in the two unimodal conditions.

Odor long-term memory is reported to be extraordinarily robust to decay (Herz and Engen, 1996). This finding is reflected in our data: Although explicit visual performance was much higher at the first time of testing, participants achieve nearly equal explicit performance in all three modalities at the second time of testing. Both, explicitly and implicitly processed odors lead to almost equal performances after 1 month whereas implicitly processed visual landmarks seem to be no longer represented in long-term memory as performance is below chance level (one third). In the multimodal condition, the landmark information of the long-term odor memory seems to be able to compensate for the loss of the implicitly processed visual information. Thus, in a real environment consisting of multimodal stimuli, we manage to achieve the best possible performance in wayfinding both explicitly and implicitly by relying on all our senses.

Furthermore, the present study as well as previous studies demonstrate the ability of humans to orient themselves using olfaction (wayfinding performance above chance level; e.g., Porter et al., 2007; Hamburger and Knauff, 2019). However, consciously we rely on the visual–auditory spatial frame for orientation. After all, even though we possess a rather well-established sense of smell, we trust our nose the least of all sensory modalities (Classen et al., 1994; Lundström et al., 2008). In general, humans not only do not report using their sense of smell for orientation, but also lack confidence in their ability to use it (Koutsoklenis and Papadopoulos, 2011; Hamburger and Knauff, 2019). This is because - while our threshold for detecting odorants is very low (e.g., Cain, 1977; Porter et al., 1983; Nagata and Yoshio, 2003) – we are only aware of unusually high odor concentrations (e.g., Lorig, 1992). However, studies have shown that humans can switch from implicit to explicit odor processing in navigation through practice, as seen in lateralization tests (Negoias et al., 2013) and scent tracking (Porter et al., 2007). Thus, it appears that our capacity for olfactory spatial processing is still intact, but it typically operates unconsciously and can be harnessed explicitly with training (Wudarczyk et al., 2016). Based on our findings and existing research, we therefore believe that the main reason for not considering the sense of smell in orientation is its largely unconscious nature and not that it is useless for human orientation.

### 4.1. Limitations and implications for future research

Although all interactions of the three-way ANOVA became significant, none of the pairwise comparisons were significant. In addition, with the experimental design at hand, it will never be possible to prove whether a landmark was processed implicitly but still led to a wrong route decision, since it can also be a mere failure in performance without the landmark having been processed at all (neither implicitly nor explicitly).

For this reason, the study provides only initial evidence. To gain a better understanding, it requires much more sophisticated designs, which we are currently working on for future studies. Nevertheless, we consider the study to be particularly relevant because it has already been able to replicate findings previously found in studies of implicit olfactory memory without a complex experimental design (Moessnang et al., 2011; Wudarczyk et al., 2016). It supports our stated hypothesis and the olfactory spatial hypothesis that a consistent pattern emerges in the few available studies with a wide variety of experimental designs (Moessnang et al., 2011; Wudarczyk et al., 2016). If a simple, commonly used design like this can replicate the data, we look forward with great confidence to the results of future research in this area.

Future studies could search online databases for further experiments using recognition and wayfinding tasks to perform analogous computations with already existing data sets in wayfinding research. The data from, e.g., Hamburger and Röser (2014) or Arena and Hamburger (2022) are suitable for re-evaluation to explore the differences between implicit and explicit processing of the auditory and olfactory sense in wayfinding. It is not always necessary to conduct new, expensive experiments to explore a new question.

Based on our data we can only suggest that the performance achieved by the participants is due to short-and long-term memory effects, different mechanisms of implicit and explicit processing between distinct modalities, and compensation of weaknesses of one modality by strengths of the other modality. However, these differences need to be further studied in the future.

## 5. Conclusion

The results suggest distinct implicit processing mechanisms within different sensory systems. The best wayfinding performance could be achieved by implicit processing of olfactory stimuli and explicit processing of visual stimuli. It supports and extends the findings of Moessnang et al. (2011) and Wudarczyk et al. (2016) on the existence of an implicit-explicit dissociation of olfactory localization. Finally, the results could lead to new insights and a better understanding of consciousness, as olfaction strongly influences our behavior, but remains largely latent unless deliberately honed through practice. With our study, we highlight the need for future studies to invent and use further experimental methods that capture implicit memory and processing from all of our sensory systems.

## Data availability statement

The raw data supporting the conclusions of this article will be made available by the authors, without undue reservation.

## Ethics statement

The studies involving humans were approved by local ethics committee of the Department of Psychology (06), Justus Liebig University Giessen. The studies were conducted in accordance with the local legislation and institutional requirements. The participants provided their written informed consent to participate in this study.

## Author contributions

MS: Writing – original draft. KH: Writing – review & editing.
