# Supplementary material for: Implicit versus explicit processing of visual, olfactory, and multimodal landmark information in human wayfinding
Source: Front Psychol. 2023 Nov 15;14:1285034. doi: 10.3389/fpsyg.2023.1285034 (PMC10684750; doi:10.3389/fpsyg.2023.1285034)

**Supplementary material**

**Implicit versus explicit processing of visual, olfactory, and multimodal landmark information in human wayfinding**

**1. Visual and Olfactory Landmarks and Distractors and Corresponding Example Pictures**

The visual landmarks were taken from private sources and the license free stock images provider pexels.com.

**Landmarks:** Fish, Salami pizza, Nail polish, Alcohol, Pineapple, Gras, Aniseed, Fresh Laundry, Eucalyptus Pepper, Curry, Citron, Vanilla, Aftershave, Tangerine, Strawberry, Basil, Cocoa


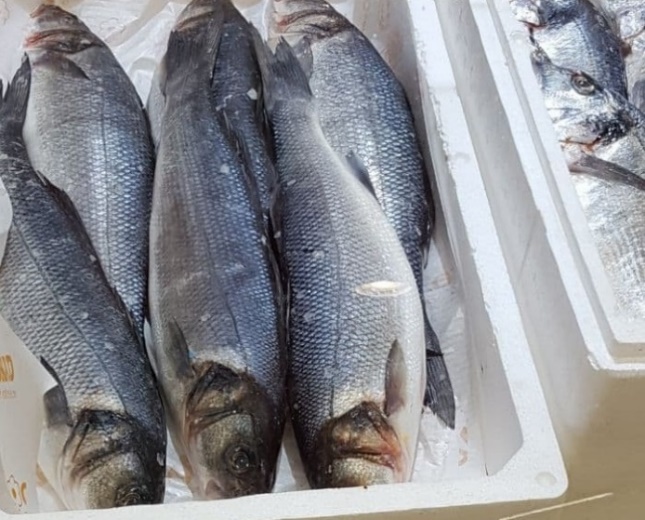

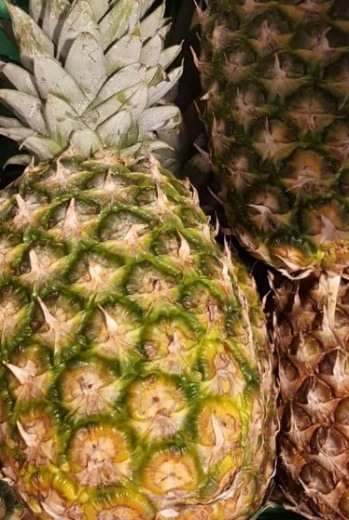

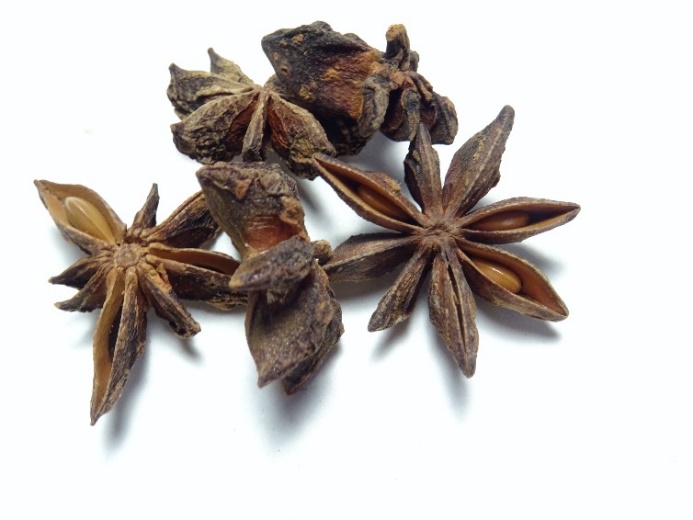

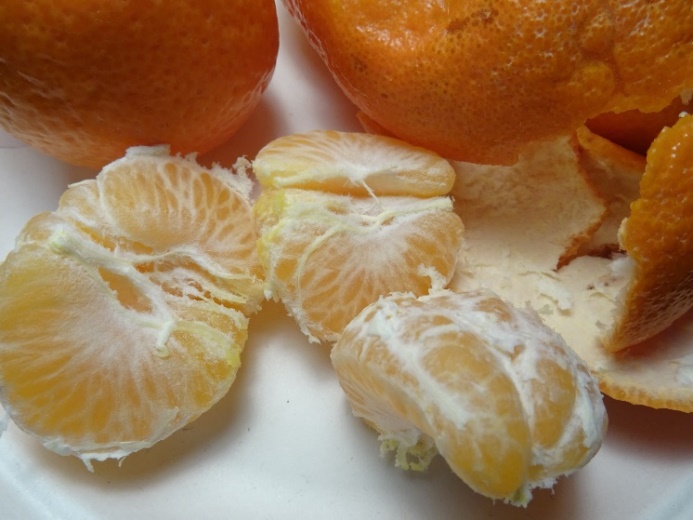

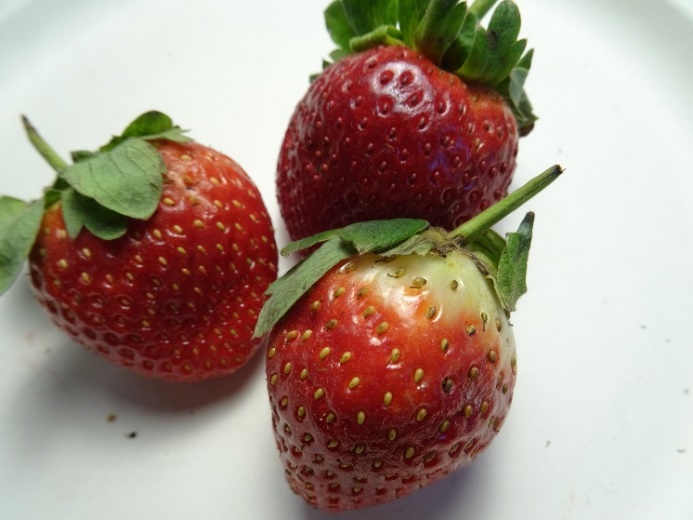


**Distractors:** Garlic, Vinegar, Leather, Spruce needle, Peanut, Frankincense, Coke, Lavender, Melon, Clove, Peppermint, Coconut, Banana, Apple, Rose, Cinnamon, Licorice, Black Tea


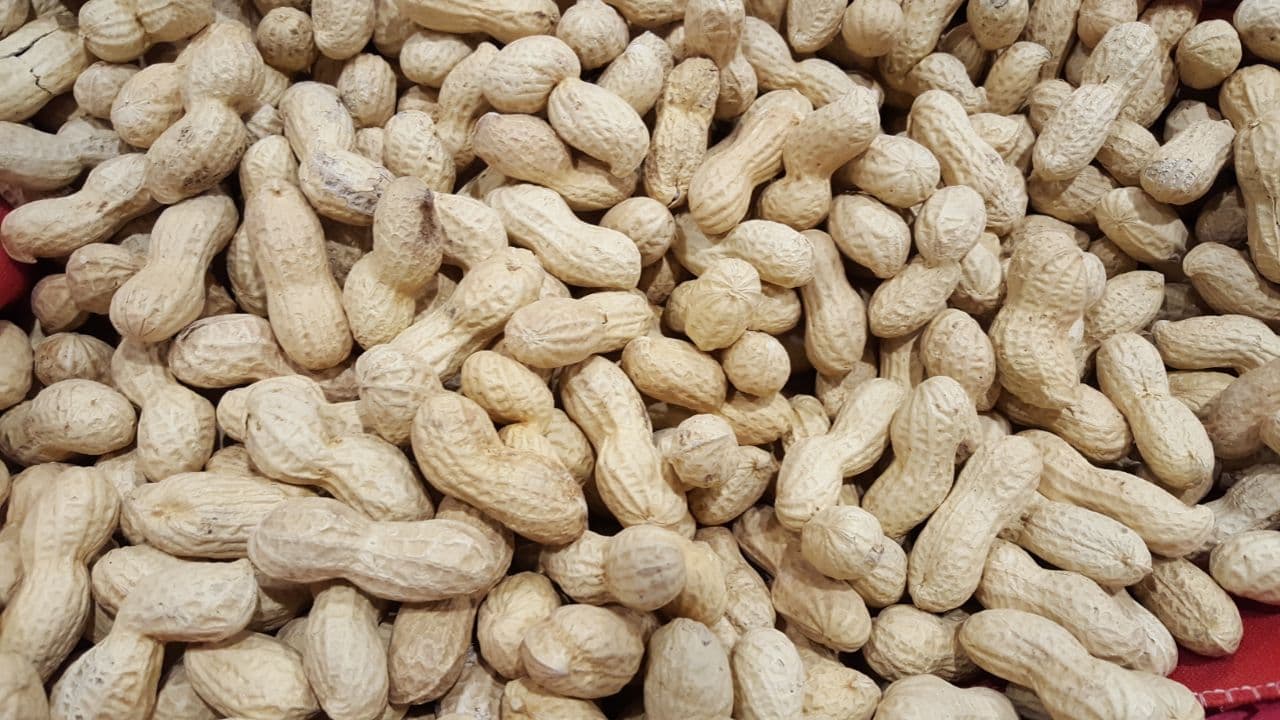

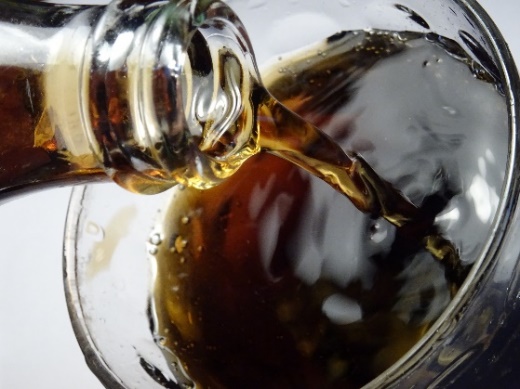

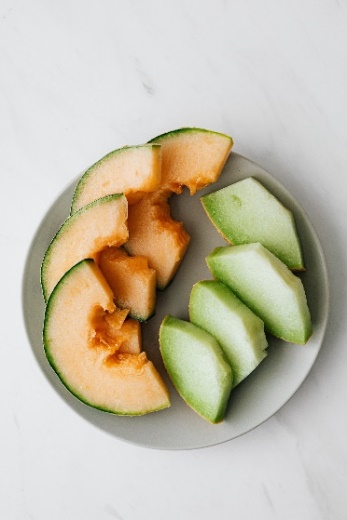

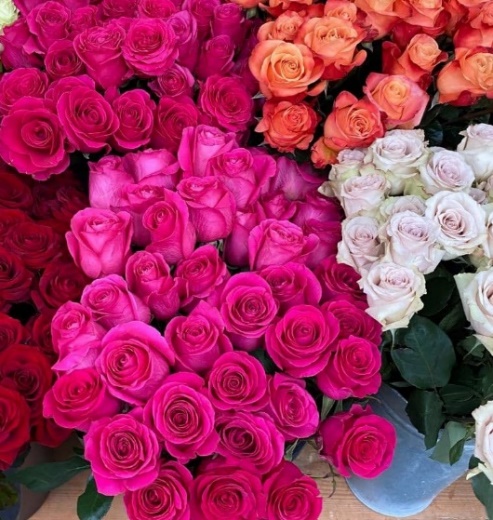

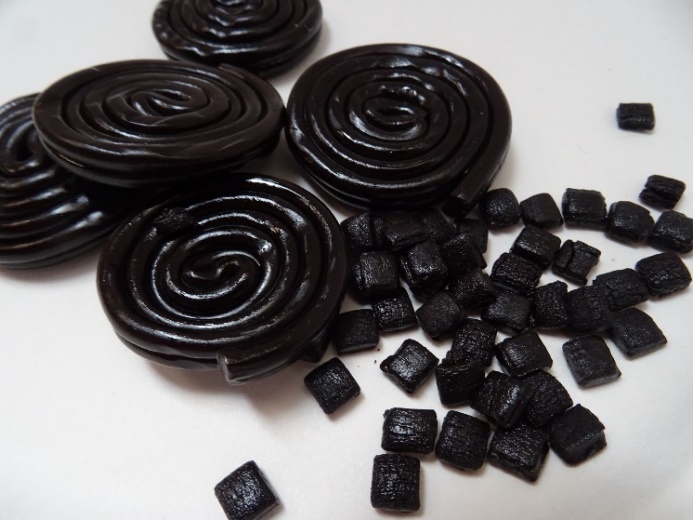

Supplement: Supplementary file 1 [file Data_Sheet_1.docx]
